# Report of a Case of Syphilis, with Remarks

**Published:** 1856-02

**Authors:** W. F. Westmoreland

**Affiliations:** Professor of Surgery in the Atlanta Medical College


### ARTICLE II.

*Report of a Case of Syphilis, with Remarks.* By W. F. WESTMORELAND, M. D., Professor of Surgery in the Atlanta Medical College.

Mr. P——, of DeKalb county, aged forty years, presented himself at the Surgical Clinic, sometime in August of the past year. The history of the case given by the patient, at my first examination, was as follows: Near four years ago, he was attacked with an affection of the throat, which he at first imagined was nothing more than the effects of cold; it, however, continued to grow worse, and at the expiration of a few months, the soft palate, tonsils, and bones of the nose were extensively involved in the disease. The ulceration of the soft parts continued to make progress until the whole of the soft palate was destroyed; the disease of the bones also increased, until several pieces of the nasal bones were eliminated—leaving the nose considerably deformed.

The following were the appearances presented at my first examination: Great deformity of the nose; complete destruction of soft palate; deep seated ulcers of the tonsils, and an extensive ulcerated surface of that portion of the pharynx attached to the bodies of the cervical vertebra. The bodies of the vertebra were also involved in the disease, the least motion of the head giving him pain.

He positively denied ever having had a chancre, or any other symptom that would indicate primary Syphilis, and from the character of the man, I do not hesitate to give full credit to his statement. Said that he had been prescribed for by a number of physicians, sometimes with apparent benefit—had prescribed for himself a length of time, and in addition, had taken $280 worth of patent medicine.

At my next examination, I was more rigid in my investigation of the case, and in addition to the above, learned the following in regard to his parents, and his extreme infancy: He said that his mother, who is still living, tells him that five or six years before he was born, his father contracted Syphilis, which proved very difficult to manage, confining him to his room for a length of time. That he, (the patient,) when an infant, was attacked with an eruption, which the physician in attendance diagnosed, and treated as Syphilitic—imagining that he had contracted it from his nurse. Under the treatment for Syphilis, the eruption in a short time disappeared, since which time, until the present attack, he has enjoyed most excellent health.

Being fully satisfied in regard to the Syphilitic origin of the disease, I at once put him on the following prescription:

℞ Syrupus Gentianœ, ℥vii; Potassii Jodidum, ʒviss. Solve.

A table-spoonful to be taken morning, noon and night in a wine-glassful of a decoction of the bark of the root of the Juglans Nigra. He also used the above decoction as a gargle two or three times in the twenty-four hours.

Under the above treatment he rapidly improved, and at the expiration of three months, the disease had entirely disappeared. He, however, still continues the same prescription in smaller doses.

*Remarks.*—Whether the above be a case of hereditary Syphilis, or, as suggested, contracted from the nurse, it goes far to sustain the doctrine so long and ably defended by M. Ricord; that when the system is once affected with constitutional syphilis, we have no assurance whatever, of ever being able to entirely eradicate it—that it may remain latent for five, ten, and even forty years, and then reappear with all its former violence. In other words, if we treat a case of constitutional Syphilis, let our treatment be what it may, and as long continued as desired, we cannot say to our patient—you need never fear a return of the disease.

In a discussion of this subject in the *Academie de Medecine*, Oct. 1853, M. Ricord, in the course of his remarks, says: "I not only admit latent Syphilis, the Syphilitic diathesis, these terms signify the same thing; but I go still further; and do not know at what point in the treatment of constitutional Syphilis, do what we may, however rational may have been the treatment employed, that we can say with certainty, that the Syphilitic diathesis is entirely removed. My opinion is, that when the Syphilitic diathesis is once established, there remains always a germ, susceptible, in certain subjects, of manifesting itself at long intervals, by the re-appearance of the disease, which we in vain attempt to attribute to a new infection. Hence, this general rule, (I do not say law, as that would appear too ambitious,) that the Syphilitic diathesis does not double—that the evolution of Syphilis never re-commences during the continuance of the Syphilitic diathesis; in other words, that one can not, generally, have constitutional Syphilis but one time. Seventy years experience at the Hospital *de Medi*, have convinced me that we can not reproduce upon the same individual the *induratic chancre*; this is also the opinion of M. Puche, he having never been able to reproduce, on the same individual, an induratic chancre. That which has been taken for a new manifestation, or for the consequences of a new infection, is nothing more, most ordinarily, than the continuation, the successive development and evolution of the first infection.

"Thus, it is understood that I admit that constitutional Syphilis once produced, the Syphilitic diathesis established, that this diathesis may persist a great while, most frequently during life; manifesting itself at longer or shorter intervals: being arrested, it again commences its march, and is again arrested to recommence at some future period, and thus during the life of the subject; that in the intervals of these manifestations the individual may present all the appearances of health. I have seen examples of these manifestations or explosions, after more than forty years had elapsed. I recollect distinctly an individual that I cured in 1848 or 1849, of tertiary Syphilis, the origin of which was a chancre, contracted in 1801.—This, then, is a fact well established, that we may, twenty, thirty and forty years after an apparent cure of Syphilis, see a reappearance of the disease."

In this connection, it is perhaps necessary to add, that in the same discussion M. Ricord contends that hereditary Syphiilis and that contracted by the individual, present the same phenomena, and are governed by the same laws.

That Mr. P.'s case was Syphilis, I think does not admit of a doubt. If the history, appearance, &c., were not sufficient to satisfy us in this regard, it yielding so readily to an appropriate treatment for tertiary syphilis, would go far to remove any doubts that we may have had as to the character of the disease.

Whether it was hereditary or acquired, I shall not pretend to say. For those who believe in the possibility of the inoculation of constitutional syphilis, it would be a case of acquired syphilis; and for those who opposed the doctrine of inoculation, among whom is M. Ricord, it would be a case of hereditary syphilis.

